# Attitudes toward Transsexuality, Empathy, and Bullying in Young Population

**DOI:** 10.3390/ijerph19073849

**Published:** 2022-03-24

**Authors:** Jesús Esteban Mora, Francisco Manuel Morales Rodríguez, Juan Pedro Martínez Ramón

**Affiliations:** 1 Department of Educational and Developmental Psychology, Faculty of Psychology, Campus Universitario de Cartuja, University of Granada, 18071 Granada, Spain; jesteban@correo.ugr.es; 2 Departament of Evolutionary and Educational Psychology, Faculty of Psychology, Mare Nostrum Campus, University of Murcia, 30100 Murcia, Spain; juanpedromartinezramon@um.es

**Keywords:** attitudes, bullying, coexistence, coexistence, education, empathy, LGBTQ+, transphobia, youth

## Abstract

Affective-sexual and gender diversity is an increasingly distinctive and extended reality and should be acknowledged and respected. From a psychosocial and educational point of view, it is appropriate to review young people’s attitudes and knowledge regarding this, relating them to aspects such as empathy, violence, or bullying, to implement quality education in the early stages of primary education. The main objective of this study was to analyze the relationship between empathy levels, attitudes toward transsexuality, and bullying among Spanish university students. The sample consisted of 247 students. Instruments were administered to evaluate negative attitudes toward transsexual people, gender ideology, transphobia, bullying, and empathy. Inverse relationships were found between transphobia and empathy. Regression analysis demonstrated the predictive ability of empathy on attitudes toward transsexual people. The results of this study are expected to increase awareness in society and encourage appropriate, satisfactory, or tolerable coexistence, in which all individuals can be free to live and express themselves. While the results indicated that the quality of life of transgender people has comparatively improved, there is still a long way to go.

## 1. Introduction

Differences and distinctions have been found in many aspects among people. These differences can manifest as interests, preferences (both voluntary and involuntary), and even ways of being and feelings. Children who are far from any social norm or prejudice do not feel the need to label and differentiate themselves from others. However, we live in a world that is full of labels and categories, and each of them is associated with different stereotypes [1].

Consequently, people begin to live with these labels since they are born. These labels rarely change and tend to tag the people who carry them. This social problem causes conflicts and difficulties in a person’s life who does not feel identified with a label that has been given involuntarily at a certain point in their life [1].

These conflicts translate into discrimination, social exclusion, school bullying, work harassment, and even irrational hatred. Primarily, we focus on people who do not identify with the gender that they were assigned at birth: transsexual or transgender (trans) people [1,2]; a substantial gap is evident between theory and reality.

To understand the entirety of this research, it is convenient to clarify the terms that are addressed. First, we begin with gender identity. Gender identity is composed of gender and sex. Thus, as stated by several authors such as [3], sex is a set of physiological, biological, and hormonal characteristics that will cause a person to be classified as a man or a woman. Gender is a social and cultural construct that is associated with different behaviors or roles that are attributed to masculinity and femininity. Importantly, gender goes beyond sex, and only the persons themselves are capable of classifying themselves as of one gender or another.

Therefore, gender identity starts the moment a person is classified with respect to sex; thereafter, the concept of gender develops when they are educated in a certain way while undergoing a distinctive and unique process of socialization [3]. Some gender-related terms are cisgender or cis (those who identify with the sex that was assigned at birth); non-binary gender (those who do not fully identify with one gender but with several or all of them at once,), and transgender/transsexual or trans (those who do not identify with the sex that was assigned at birth). The theoretical and scientific basis that supports children’s identity formation is Bandura’s social learning theory [4]. It considers that the acquisition of gender roles, as well as aggressive and discriminatory behaviors toward this group, constitute a process of vicarious learning or that which is gained through observation. Likewise, it can be pointed out that negative attitudes toward gender equality and stereotypes can translate into a greater predisposition toward harassment, discrimination, and violence against lesbian, gay, bisexual, transgender, queer, intersex, asexual, and plus (LGBTQIA+) individuals [5,6,7], which has a negative impact on their self-esteem [8].

Moreover, it is relevant to develop the concept of sexual identities or orientations; “sexual orientation is an enduring emotional, romantic, sexual, or affectional attraction to others” [9] (American Psychological Association, Worcester, MA, USA, 2013, p. 1). Some sexual orientations are defined as follows: bisexuality (a person’s emotional, romantic, sexual, or affectionate attraction to people of their own and other genders); heterosexuality (a person’s emotional, romantic, sexual, or affectionate attraction toward people of the opposite gender); and homosexuality (a person’s emotional, romantic, sexual, or affectionate attraction toward people of their own gender).

Another important concept is that of transphobia, which refers to the negative attitudes that are directed at people whose gender does not correspond to the sex that was assigned at birth. These attitudes include hate, aversion, and fear [10].

To consider the importance of the representation of the lesbian, gay, bisexual, transsexual, queer, and plus (LGBTQ+) community in primary education, it is important to discuss the Spanish legislation in relation to the protection of this community. The state level is composed of Law 13/2005 [11], which grants equal marriage and Law 3/2007 [12], which in turn regulates the change of name and sex in the National Identity Card. In addition, in June 2021, the Council of Ministers approved the Draft Bill for the real and effective equality of transgender people and to guarantee the rights of the LGBTQ+ community. This law deals with issues such as gender self-determination, thus eliminating the requirement of two years of hormone treatment or medical reports when modifying the ID card.

At the regional level, only eight regions have legislations protecting the rights of transgender people: Andalucía, Aragón, Islas Baleares, Islas Canarias, Madrid, Navarra, Comunidad Valenciana, and País Vasco. A total of 10 have legislations that protect from discrimination against the LGBTQ+ community: Andalucía, Aragón, Islas Baleares, Cataluña, Extremadura, Galicia, Madrid, Murcia, Navarra, and Comunidad Valenciana. Finally, five communities do not have any law protecting this group: Asturias, Cantabria, Castilla y León, Castilla-La Mancha, and La Rioja.

Various international, national, autonomous, and local legislations are responsible for implementing protocols and guidelines that are aimed at protecting LGBTQIA+ individuals and educating others about them. One recent and relevant example is that of UNESCO’s contributions to the LGBTQIA+ community through its technical advice to states and researchers to explore and measure school violence and resilience among LGBTQIA+ youth [13]. Another example was the research training support that was provided by the organization to states and researchers to explore resilience for the LGBTQIA+ community [14]. These worldwide contributions of UNESCO are considered relevant for psychoeducational assessment and intervention by enabling the monitoring of school violence based on sexual orientation, gender identity or expression, and group resilience. This may be important in preventing school violence and promoting its evaluation in the classroom—a key aspect in tackling situations of violence and bullying, according to [15].

At the national level in Spain, since the implementation of the Organic Law of Education in 2006 [16], a regulatory framework was established to improve coexistence in educational centers, promote respect and tolerance, and encourage value for student diversity. At its core, the Organic Law 3/2007 of 22 March [17] is a framework for the effective equality of men and women. Regarding the educational field, it states that it will be in charge of the following: (a) suppressing educational materials of the existence of sexist stereotypes, (b) including the principles of equal treatment in the different stages of the educational system, (c) teacher training in the subject of equality in continuous training and the study plans of the degrees, (d) cooperation between administrations to promote equality and non-discrimination, and (e) implementing measures for the recognition of women in history.

Since this state law was passed, autonomous communities in Spain have adopted their own laws to include different areas, such as the educational field, in the development of their plans to encourage coexistence. Practically, all autonomous communities have protocols for action in cases of homophobic or transphobic bullying in educational centers. For example, in Andalusia [18], a specific document was published by the Ministry of Education in this regard, outlining guidelines for appropriate educational attention to students according to their gender identity. Another pioneering region, the Canary Islands [19], updated a document entitled “Protocol for the accompaniment of transgender students and attention to gender diversity in schools in the Canary Islands” in 2017. For example, in the Balearic Islands [20], a coexistence plan for educational centers through a “Trans Protocol for transsexual and transgender students” attempted to guarantee access to facilities such as toilets and changing rooms according to individuals’ gender identity. Further, notably, in the plan for attention to diversity as a tool for equality in the region of Murcia [21], general, ordinary, and specific measures were contemplated as a prescriptive document for public centers. More recently, in Law 8/2017 of 28 December, to guarantee the rights, equal treatment, and non-discrimination of LGBTQIA+ individuals and their families in Andalusia [22], measures were contemplated to combat school harassment in Article 14. In particular, referring to the university environment, it promoted training and research measures and a clear commitment against discriminatory attitudes stemming from LGBTQIA+ phobia in Article 17.

Considering the care and protection of the LGBTQ+ community in Spain, it is important to consider the implementation of the relevant laws in the domain of education, among students.

It is of significant importance to work on the gender stereotypes in educational institutions and the students’ immediate environment. For this reason, it is necessary to make use of socio-educational models that are based on empathy and respect [23,24]. The connection between the two concepts derives from the fact that empathy entails putting oneself in the other person’s shoes, which is an essential condition for showing respect.

Several studies [1,2] discuss how the mere fact of belonging to the LGBTQ+ community, specifically being a transgender, is an additional risk when it comes to becoming a victim of bullying or school violence. However, Ref. [25] talked about the pedagogy of containment, in which schools try to restrict and contain knowledge about affective-sexual and gender diversity to manage the risks of putting such issues on the table. Dealing with this information in an informative manner and creating a safe space in schools that sets a precedent is retained with the aim that the identified student does not suffer the consequence of being in the spotlight. This approach is not seen as problematic, although it is at odds with visibility and inclusion.

A study that was conducted in Spain [26] found that 24.5% of the children that were interviewed were victims of transphobic bullying. Of that percentage, 92.3% thought it was related to their gender dysphoria. For this reason, it is necessary to work with all students, with the aim of creating an environment of coexistence in which all parties can fully develop.

Another study that was conducted in Spain by Pérez et al. [27] in 2021, compared the bullying of cisgender people with the bullying of transgender people at school. The study found no major differences between the groups because most of the transgender students interviewed did not publicly express their gender identity. In addition, all the participants received psychological support. Similar to other studies [28,29,30,31], the aggressor is usually a male of the same age or older than the victim.

Presently, an increasing number of studies have analyzed young people’s attitudes toward affective-sexual and gender diversity [32,33], although more research is needed. This may be due to the wide variability of sexual behaviors arising in an environment of freedom.

Another study that talks about the relationship between some of the variables in the current study is that of Nolasco [34], in which he relates empathy with bullying. His conclusions explain that women tend to be more empathetic than men (something that can change if we talk about gender) and that less empathetic people are more likely to experience violent behavior in the school environment and, therefore, commit bullying.

Therefore, the main variables of the current study were transphobia, empathy, and bullying. As previously defined, transphobia refers to negative attitudes (hate, aversion, and fear) that are directed at people whose gender does not correspond to the sex that was assigned at birth [10]. Empathy corresponds to the ability to understand and feel the emotions of others [35]. Bullying is a repeated aggressive behavior that interferes with social and moral norms, as these acts are intentional and include denigrating elements for the victim [36].

Further references on the three study variables—empathy, bullying, and transgender people—are shown below.

A recent study [37] on the relationships between homophobic attitudes toward gender-based partners in young people found that empathy was a strong predictor of such attitudes. It also found moral disengagement to be a predictor of aggressive behavior toward such individuals. Another study [38] sampled 282 students aged 18–24 years, mostly of Caucasian origin, in a private university in Southern California. It found that empathy positively predicted openness to diversity-encompassing aspects such as those referring to sexual orientation. Further, a study [39] found that participatory theater interventions to reduce discrimination and stigma affecting LGBTQIA+ individuals increased empathy and understanding of them, indicating a change in attitude and awareness toward them. Another study [40] on the improvement of care for transgender people in the Doctor of Pharmacy Curriculum included, among the contents of the educational materials, precise considerations that were related to empathy, in addition to other medical and cultural considerations. Moreover, another study [41] evidenced that increasing the average number of intergroup contacts with transgender people increased empathy toward them and decreased possible existing biases in this regard.

Regarding bullying, Earnshaw’s [42] work noted that lesbian, gay, bisexual, and transgender youth report verbal, relational, and physical harassment and damage to property, along with various effects on their well-being and physical and mental health. To address this issue, recent research [43] indicated that the promotion of LGBTQIA-inclusive sexuality education attitudes and the development of interventions that foster a positive school climate could reduce victimization and bullying that is experienced by sexual minority youth. Further, it could lessen adverse mental health symptomatology that is related to depression and suicidal tendencies. Trans individuals living in gender-segregated environments experience more negative attitudes, intolerance, discrimination, and barriers [44]. This makes the assessment of such attitudes more difficult because, as highlighted by [45], instruments that capture the full diversity of non-traditional gender role attitudes and not only the predominant ones from a binary model are required.

A recent study [46] found that sexual minority youth were more likely to report experiencing sexual and physical intimate partner violence compared to heterosexual youth. Likewise, schools with more affirming climates for LGBTQIA+ students reduced the likelihood of reporting physical, but not sexual, intimate partner violence for female students. Instead, they found that LGBTQIA-affirming school climates increased the risk of sexual intimate partner violence for gay male students. These authors [46] concluded by emphasizing the need for more in-depth studies with an eye toward sexual violence prevention. Similarly, another recent study [47] found that the highest risk of intimate partner violence was in transgender youth (especially those who are transgender and lesbian, gay, bisexual or who are transgender and question their sexual orientation). They also found relationships between intimate partner violence, bullying victimization, online bullying victimization, and mental health (depressive symptoms and suicidal tendencies).

Another study [48] focused on examining how certain heteronormative variables affect violence against trans individuals, among other aspects. The study found that a higher level of hostile heteronormative sexist and racist attitudes, as well as a lower endorsement of expressive traits (and a higher endorsement of instrumental traits), were predictor variables of a higher level of violence toward trans and gender-diverse individuals in the student body. A previous study [49] found the existence of negative discriminatory attitudes toward trans individuals in both an affective/cognitive dimension and a behavioral dimension, being more negative in adolescents compared to adolescent girls. Other research [50] has also found that identification as lesbian, gay, bisexual, transgender, or queer is a significant sociodemographic predictor of the variable suicidal ideation and that increasing the development of empathy was one way to reduce such suicidal ideation.

However, notably, the data may not be considered entirely conclusive, and further research is needed on variables that may influence violent behaviors and negative attitudes toward trans individuals.

For this reason, this study aimed to analyze the negative attitudes or discrimination toward transgender people and their relationship with different psychoeducational variables such as empathy and the relationship with other variables, such as bullying. This study focused on analyzing the relationship between empathy and transphobia. It was hypothesized that both variables have an inverse relationship; that is, if a person scores high on empathy, their transphobia variable score will be low, and vice versa. Further, this study aimed to analyze the relationship between bullying and transphobia. It was hypothesized that both variables have a direct relationship, that is, if a person scores high on bullying, their transphobia variable score will be high and vice versa.

## 2. Methods

### 2.1. Objectives and Research Hypotheses

This study aimed to analyze the self-reported levels of negative attitudes toward trans individuals (transphobia) and its relationship with empathy and bullying in a sample of young people. Further, the study intended to determine if empathy and bullying are related to the levels of transphobia in this population.

**Hypotheses** **1** **(H1).** *The research hypotheses of this study are as follows. First, the self-reported levels of negative attitudes toward trans individuals (transphobia) are related to empathy in the higher education context—the higher the levels of transphobia, the lower the levels of empathy.*

**Hypotheses** **2** **(H2).** *Second, the levels of negative attitudes toward trans individuals (transphobia) are related to bullying—the higher the levels of transphobia, the higher the levels of bullying.*

### 2.2. Participants

The overall sample consisted of 250 university students from three academic areas—humanities, social, and health sciences—in the University of Granada, Granada, Spain. The final sample comprised of 247 university students. About 63% were women; most of them were first- and second-year students that were aged between 18 and 23 years. We used an incidental sample. There were no differences between the academic areas according to sex and age. Part-time students, students with special educational needs, and students with incomplete questionnaires were excluded from the final sample. This was done to narrow down the population to make further comparative studies more feasible. We calculated the sample size that was required to detect this size effect in the sample. The calculation was carried out using G*power 3.1 software (version 3.1, Institut für Experimentelle Psychologie, Düsseldorf, Germany). It indicated that a sample size of 145 university students was needed to provide a confidence interval of 95%, with a power of 95%, assuming a bilateral significance level (α) of 0.05.

### 2.3. Instruments

#### 2.3.1. Scale of Negative Attitudes toward Trans People

This scale [33] consisted of nine items. It was used to evaluate the expression of prejudice toward these individuals according to the degree of agreement with the statements that were presented. The total score was obtained by adding the scores based on a 5-point Likert scale ranging from 1 = strongly disagree to 5 = strongly agree. This scale has a good, almost excellent internal consistency (α = 0.886). Higher scores are indicative of a higher level of negative attitudes toward transgender people.

Some items in this instrument are: “*Trans people should not be allowed to teach in schools*; *“ ”trans people tend to be sexually promiscuous; “ ”trans people are more likely than the rest of society to contract a sexual disease*”.

#### 2.3.2. Gender Ideology and Transphobia Scale

This scale [10] consisted of 12 items. It was answered with a Likert-type scale ranging from 1 to 7. The total score was obtained by adding the scores based on a 7-point Likert scale ranging from 1 = strongly disagree to 7 = strongly agree. This scale has good, almost excellent internal consistency (α = 0.88). Higher scores are indicative of a higher level of negative attitudes toward the transgender people.

Some items in this instrument are: *“If I saw a man on the street whom I suspected was a woman I would ask him his sex; “ ”God made two sexes and only two sexes; “ ”It is immoral for a woman to present herself in public as a man”*.

#### 2.3.3. Transsexuality Attitudes Scale

This scale [32] consisted of nine items. The person that was being evaluated was asked about the degree of agreement with a series of statements. The total score was obtained by adding the scores based on a 7-point Likert scale ranging from 1 = strongly disagree to 7 = strongly agree. This scale has acceptable, almost good internal consistency (α = 0.762). Higher scores are indicative of higher level of negative attitudes toward transgender people.

Some items in this instrument are: *“I don’t like it when someone is flirting with me and I can’t tell if it is a man or a woman; “ ”I would be upset if someone I have known for a long time revealed to me that he/she used to be a different gender; “ ”I feel uncomfortable around people who do not conform to traditional gender roles, for example, aggressive women or emotional men”*.

#### 2.3.4. Cognitive and Affective Empathy Test

This scale [51] consisted of 33 items. The person that was being evaluated was asked to choose between “strongly disagree, agree, undecided, disagree, and strongly disagree” with the statement indicated in each item. The total score was obtained by the sum of scores based on a 5-point Likert scale ranging from 1 = strongly disagree to 5 = strongly agree. The cognitive dimension includes the dimensions of perspective taking (ability to tolerate, communicate, and relate) and emotional understanding (ability to know and understand others’ emotional states, intentions, and impressions), while the emotional dimension includes empathic stress (connection with others’ negative emotional states) and empathic joy (ability to share others’ positive emotions). This scale has good internal consistency (α = 0.77–α = 0.86).

Some of the items in this instrument are: *“When a friend is sad, I get sad too; “ ”I rarely do not recognize how a person close to me feels just by looking at him/her; “ ”When a friend has behaved badly toward me, I try to understand the reasons why”.*

#### 2.3.5. European Bullying Intervention Project Questionnaire (EBIPQ)

This scale [52] consisted of 14 items. The total score was obtained by the sum of scores based on a 5-point Likert scale ranging from 1 = strongly disagree to 5 = strongly agree. This scale has acceptable, almost good internal consistency (α = 0.785). Higher scores indicate higher level of evidence of bullying. For this, the person that was being evaluated was asked about possible experiences related to bullying in their environment (school, friends, acquaintances) as a victim and/or aggressor; questions such as *“Have you experienced any of the following situations in the last year?”* were posed.

Some items in this instrument read as follows: *“Someone has hit, kicked, or pushed me; “ ”someone has insulted me; “ ”I have been excluded or ignored by others”*.

At the end of all the instruments, the following open question was asked: “*Finally, would you like to include a reflection on the topic?*” This was only analyzed from a reflective perspective and is considered useful for other pedagogical debates that can be generated from the educational classrooms.

### 2.4. Procedure

Questionnaires were completed in class groups at the Faculty of Educational Sciences and Psychology of the University of Granada. The study participants’ complete anonymity was ensured, as they were not required to provide any identifying data (full name or ID) but were required to sign the informed consent form before completing the questionnaire. The study adheres the principles established in international and national legislation in the fields of biomedicine, biotechnology, and bioethics, as well as all the rights derived from the protection of personal data. The total time taken to complete each questionnaire was approximately seven minutes. The study obtained a favorable report from the Ethics Committee of the University of Granada (2056/CEIH/2021).

### 2.5. Data Analysis

Once the data were obtained, statistical analysis was performed, in which an ex post facto design was used. The analysis was conducted using the SPSS 22.0 computerized statistical package. Exploration of the study variables was conducted (descriptive statistics and Kolmogorov–Smirnov normality tests); compliance with the assumptions of normality and non-collinearity for Pearson’s correlation was ensured. Descriptive analyses and analyses of the relationships among the quantitative variables were performed using Pearson’s correlation analysis and linear regression.

## 3. Results

### 3.1. Descriptive Statistics

The final sample consisted of 247 university students; 63% were female, and most were between 18 and 23 years of age. We used an incidental sample in this study.

With respect to sexual orientation, 67.2% of the participants considered themselves heterosexual, 17.6% were bisexual, 7.2% homosexual, and 2.4% considered themselves to be demisexual.

The percentages that related to their sexual identity were as follows: 61.2% were cis female, 35.5% cis male, 1.7% non-binary, and 1.7% unspecified.

Based on place of birth, 72% were born in Andalusia, while the remaining 28% were born in another region or even in a country other than Spain. The majority indicated the region of Andalusia as their place of residence, although there were participants from other regions such as Alicante, Murcia, and Valencia following that order.

Table 1 shows the descriptive statistics for the variables of the negative attitudes toward transgender people, gender ideology, and transphobia; attitudes toward transsexuality, cognitive, and affective empathy; and bullying.

The results of these descriptive analyses revealed that the sample demonstrated less transphobic attitudes and more in favor of gender ideology. An average score of 11.03 was obtained in this study.

As for the gender ideology and transphobia scale by Hill and Willoughby [4], the average score that was obtained in this study was lower, with an average score of 15.81.

Finally, the transgender attitudes scale [17] indicated an average score of 14.86, showing that the average score in this study was lower.

For the empathy variable, the mean ranged from 27.20 for the empathic stress (emotional dimension) dimension to 39.18 for the empathic joy (emotional dimension) dimension, with the mean for the total score at 130.57.

For the variable bullying or harassment, the mean score was 28.36.

### 3.2. Relationship between Negative Attitudes toward Trans Individuals and Empathy and Bullying among University Students

Table 2 presents the relationships between the variable negative attitudes toward transsexual behavior and the variables of empathy and bullying among university students.

The results indicated the existence of statistically significant inverse correlations between empathy and negative attitudes toward transgender people, transsexuality, gender ideology, and transphobia. However, no statistically significant relationships were found between the variables of negative attitudes toward transgender people, transsexuality, gender ideology and transphobia, and variable bullying.

The total scores for empathy, emotional understanding, and empathic joy were significantly related to the dependent variable, predicting 17.8% of the total variance (*R*^2^ = 0.178, *F*(6081) = 6.081, *p* < 0.001) of the levels of negative attitudes toward transsexuality (Table 3).

At the end of each questionnaire, there was a section where the participants could leave their impressions or conclusions in the form of qualitative comments on the subject that was treated. Most of the reflections centered on the importance of the subject matter and reflected on the need to educate based on empathy and respect. Some of these comments have been presented here. Only 19.04% of the sample (10.88% of women and 8.16% of men) considered it necessary to make some reflection or comment at the end of the questionnaires. These reflexive narratives were included in the article as they show the need and appreciation of the students toward including this type of evaluation and research in an academic environment. Similarly, this reflection is considered interesting because, as was pointed out earlier, it is still necessary to construct and validate new, more inclusive instruments to evaluate negative attitudes toward transsexuality.

*“I think it is very necessary for everyone to do this questionnaire. I think you realize the things you do wrong or right in front of other people and that makes you know yourself more psychologically and makes you change those negative attitudes”.*

*“From my point of view, I think it is very important to work on these issues at school in order to have a good social coexistence with other people. In education, it should be compulsory to make issues such as racism, xenophobia, sexual and gender diversity … known to all students”.*

*“I think many parents still think in an old way, and therefore do not show empathy for homosexual or transgender people. These thoughts are transferred to their children and so on. Also, I think that in school, they don’t make these issues known and they are very necessary because then there is confusion, and they are not well informed. However, every day these issues are becoming more normalized and, although there is still a long way to go, I hope that one day everything will be normalized”.*

A reflection on the effectiveness of the instruments that were used to assess the variables that were selected for this study indicated that the number of instruments with an inclusive perspective is scarce, which leads to the need to update many of the tools that are currently used and develop new ones that are adapted to the current reality.

## 4. Discussion

The objective of this work was to analyze transphobia and other stereotypes that are related to gender identity and determine the relationship between empathy and tolerance toward affective-sexual and gender diversity.

Although the results that were obtained were consistent with previous studies [32,33], they reveal that certain stereotypes and attitudes that are seen in these studies have been overcome, as indicated by the lower percentages of transphobia and stereotypes. In the research that was conducted by [33] for the construction and validation of the scale of negative attitudes toward trans individuals, several studies were conducted in which higher levels of transphobia or negative attitudes toward trans individuals were evidenced.

These results are congruent with another study [53], which also found that negative attitudes toward trans individuals were prevalent in many situations, but higher education students had relatively more positive attitudes toward them. Similar to this study, we believe that it is necessary to increase knowledge about affective-sexual diversity and trans individuals in the university environment through actions and programs that are designed for this purpose, applying methodologies to increase positive attitudes towards trans individuals, such as that which was conducted by Gorrotxategi et al. [54].

It can be seen from the results that the responses that were obtained in this study were favorable and the population seems to lack a negative attitude toward transsexuality. The simplest conclusion would be that the population has left behind stereotypes and transphobia and is attempting to understand and respect this group. However, this was not the case. Although the percentage may seem low, we are still referring to people. Any percentage of transphobia is detrimental. As mentioned earlier, these stereotypes can translate into violence and discrimination toward students. As García-Acosta et al. [55] indicated, more interventions are required for the elimination of negative attitudes and prejudices toward trans individuals as such attitudes can translate into discriminatory behaviors toward them. Similarly, another recent study from the same year [48] highlights the need for inclusive and comprehensive LGBTQIA+ sexuality education for the prevention of sexual diversity violence. A study by Panigrahi [56] highlights the mental identification of trans individuals with the levels of empathy of an alternative gender, indicating that gender identification from birth correlates with the level of empathy.

The results showed that an inverse correspondence between the level of empathy that a person possesses and the transphobic attitudes that they develop. As the development of empathy, solidarity, and coexistence helps promote peaceful resolution of conflicts through dialogue [57], it is possible to affirm that educating based on empathy from an early age would reduce transphobia and stereotypes and contribute to the complete development of an individual. It is possible and advisable to apply this method to all diversity that can be found in a group class. Empathy, especially cognitive empathy, has been considered a relevant variable for the prediction of gender violence [58], with its presence being necessary to develop it. Other research [59] shows that education for the development of empathy can be an effective way to prevent different types of violence. Similarly, a more recent study [50] points out the importance of empathic functioning to reduce suicidal ideation, which is a risk factor in this population.

Many studies show that designing educational activities and actions that promote empathy, positive attitudes, and contacts with trans individuals can be an effective prevention coping strategy. For example, in the study by MacNamara et al. [60], an activity in which the gender pronouns were reversed was conducted in the classroom. The study reflections were published in an online discussion forum that allowed the promotion of empathy for trans individuals by cisgender students.

Regarding bullying, several studies [1,2,5] showed that gender identity is a risk that influences bullying and/or violence at school. This bullying is characterized by teasing, insults, and physical aggression. [25] recounted the experiences of bullied transgender students as well as their parents. Regarding the pedagogy of containment, it is important to reflect on the protection of identified students since such students will always be likely to be questioned and have to suffer the consequences of existing in today’s society. Other studies [1,2] have discussed the consequences affecting the well-being of victims, such as decreased confidence, decreased ability to trust the people around them, and suicidal thoughts. Regarding the prevention of gender-based violence and harassment, there are still only a few existing programs (even less in terms of prevention in the case of transgender people), with empathy being a key variable as considered in this study. In the implementation of programs for the prevention of intimate partner violence such as that by Garzón-Segura and Carcedo-González [61], affective empathy scores increased after the intervention, and gender stereotypes and acceptance of aggression also decreased, among other aspects. Another recent work [62] for the prevention of violence in young people focused on exposure to prosocial video games to increase empathy, such as the video game “Jesse”, which was aimed at increasing affective and cognitive empathy toward victims of intimate partner violence.

The data that were obtained in this study are considered relevant for the design of future training programs in which, as indicated by Sekoni et al. [63], it is necessary to advance a conceptual model in terms of methodology, contents, and duration of the training.

Research on the attitudes of exclusion, bullying, and perceptions of students toward gender equality in young people is necessary to expand understanding and optimize the design of primary prevention programs for school violence and negative attitudes and stereotypes in children’s education. Although recent research shows that belonging to the LBTQIA+ collective can be a risk factor for becoming a victim of bullying or school violence [2], socio-educational and community programs to attend to affective-sexual diversity are still lacking [64]. In this context, studies such as the current one are necessary to evaluate negative attitudes and prejudices toward transsexuality and its relationship with other psycho-educational variables.

However, this study has some limitations that are linked to the subject matter, since although affective-sexual and gender diversity is an issue that is becoming increasingly important in our society, there is still a lack of articles, studies, or precise information that discusses or deals with this subject. In contrast, given the specificity of the sample, it is necessary to be cautious when generalizing the results. Research results are limited in that the study involved a single university and focused only on young people whose memories of childhood and adolescence may be demobilized over past experiences in the case of cisgendered people.

In addition, it is also worth highlighting a very relevant aspect when dealing with issues that are related to inclusion. It is of significant importance to use more inclusive language and terminology so that all feel identified and included. One example are the questionnaires, which, although validated, do not use language as inclusive as they should. This also applies to the items, as some of them seem outdated and obsolete. This idea was highlighted during the survey in the section where the participants provided their views on the subject. The view of one of the participants is provided below.

*“I would like to clarify that in the answers about attraction (flirting and so on) I find it important to know the gender of the person, because I am a lesbian, and I am very uncomfortable with men. If those questions were aimed at the trans issue, I think they can lead to confusion and could be better phrased with even more inclusive language”.*

Future research should evaluate the constructs that were analyzed in this study by applying questionnaires that use even more inclusive language as well as a longitudinal design. The cross-sectional design did not allow us to establish causal relationships between the variables. Additionally, the instruments that were administered are self-report measures; the responses of the participants might have been influenced by subjectivity and social desirability. In-depth interviews will need to be conducted in another study. Future research should use more robust designs and investigate the role of other variables and factors in-depth, e.g., the role of bystanders in bullying. This will allow for the design of more specific and effective training actions to develop empathy and prevent violence against university students.

Finally, a series of proposals and reflections on the theme of the work are presented below. First, it is important for teachers to deal with affective-sexual and gender diversity from the earliest stages of compulsory education and develop and promote this subject. This is important because the topic concerns people, coexistence, and society.

Second, the role that is played by the previous and/or permanent training of the teachers on this subject is worth highlighting. Obsolete, disrespectful, or the lack of any information will lead to the marginalization of the group and spread misinformation that will contribute to transphobic and violent behavior toward this group. It is crucial for teachers to transfer a positive and empathetic attitude to students for avoiding such conflicts, and build the students to be supportive of those around them. Ref. [25] described in their research how schools tend to focus on informing students, teachers, and families about the existence of transgender students in a superficial and careless manner. They limit themselves to trying to accept and integrate them instead of talking and informing about, for example, the differences between gender identity and sex that is assigned at birth.

In addition, teachers need to be aware of the relevant regulations as well as the existing protocols of action when there is a case of violence or bullying or bullying to gender identity. In Andalusia, there is a specific action protocol on this subject since 2015, although, as mentioned earlier, there is no legislation regulating these cases or aggressions at the state level. An example of this is the article by Ferfolja and Ullman [25], mentioned earlier, in which one of the testimonies of a parent who visited the school in a case of bullying, was informed that the protocol for action was that the victim should raise their hands and say “enough”. As the episodes of violence and aggression did not stop, the parent decided to change the child’s school. This episode is an example of the importance of having an efficient, effective, and compliant action protocol, as well as a competent and empathetic teaching team that considers the needs of all types of students.

Finally, it is important to highlight the importance of ridding taboos when dealing with this subject in schools, as this is the reason students do not receive quality affective-sexual and gender education to be able to face and relate to the present society full of diversity. If students can learn to write or add, they can also learn that all people are equally important and that it is necessary to respect them all by embracing diversity.

## 5. Conclusions

In conclusion, this study found significant relationships between empathy and a positive and tolerant attitude toward affective-sexual and gender diversity. Empathy is relevant for the design of future actions, interventions, and programs concerning affective-sexual and gender diversity. Hence, it is necessary to continue evolving and educating our society to be respectful and tolerant toward all types of diversity in order to build a better world for all.

## Figures and Tables

**Table 1:** Descriptive statistics of the negative attitudes toward transgender people, gender ideology and transphobia; attitudes toward transsexuality, cognitive and affective empathy; and bullying in university students.

Variables	Min	Max	*Media*	*Standard Deviation*
**Negative attitudes toward transgender people**	9.00	26.00	11.03	3.49
**Gender ideology and transphobia**	12.00	34.00	15.81	5.63
**Attitudes toward transsexuality**	9.00	43.00	14.86	7.84
**Empathy (total)**	92.00	165.00	130.57	15.44
Perspective taking (cognitive dimension)	24.00	40.00	32.62	4.00
Emotional understanding (cognitive dimension)	19.00	50.00	39.18	5.36
Empathic stress (emotional dimension)	11.00	40.00	27.20	6.56
Empathic joy (emotional dimension)	20.00	35.00	31.59	3.64
**Bullying**	14.00	60.00	28.36	9.50

**Table 2:** Correlations between the negative attitudes toward transgender people, gender ideology and transphobia; attitudes toward transsexuality, cognitive and affective empathy; and bullying in university students.

Variables	Negative Attitudes toward Transgender People	Gender Ideology and Transphobia	Attitudes toward Transsexuality
Empathy (total)	−0.308 **	−0.276 **	−0.253 **
Adoption of perspectives	−0.291 **	−0.179	−0.244 **
Emotional understanding	0.135	−0.171	−0.129
Empathic stress	−0.370 **	−0.329 **	−0.280 **
Empathetic joy	−0.118	−0.129	−0.112
Bullying	−0.035	0.140	0.072

** *p* < 0.001.

**Table 3:** Regression of the “negative attitudes toward transsexuality” from the variables of cognitive and emotional empathy and bullying in university students.

Independent Variables	B	95% CI		*β*	*SE*	*p*-Value
		Lower Limit	Limit Top			
Emotional understanding (cognitive dimension)	0.72	0.37	1.06	0.58	0.17	0.00
Empathic joy (emotional dimension)	−0.49	−0.97	−0.01	−0.27	0.23	0.04
Empathy (total score)	0.49	0.234	0.743	0.41	0.12	0.00

*R*^2^, regression coefficient of determination; B: estimators of the regression coefficients; *β*: estimators of the typed regression coefficients; 95% confidence interval for B; *β*: adjusted coefficient of the multiple linear regression analysis; *SE* coefficient standard error; *p*: critical significance level.

## Data Availability

The data can be requested by the scientific community under the ethical terms to be determined.
